# PASS2.7: a database containing structure-based sequence alignments and associated features of protein domain superfamilies from SCOPe

**DOI:** 10.1093/database/baac025

**Published:** 2022-04-12

**Authors:** Teerna Bhattacharyya, Soumya Nayak, Smit Goswami, Vasundhara Gadiyaram, Oommen K Mathew, Ramanathan Sowdhamini

**Affiliations:** National Centre for Biological Sciences (TIFR), Bellary Road, Bangalore, Karnataka 560065, India; National Centre for Biological Sciences (TIFR), Bellary Road, Bangalore, Karnataka 560065, India; National Centre for Biological Sciences (TIFR), Bellary Road, Bangalore, Karnataka 560065, India; National Centre for Biological Sciences (TIFR), Bellary Road, Bangalore, Karnataka 560065, India; National Centre for Biological Sciences (TIFR), Bellary Road, Bangalore, Karnataka 560065, India; National Centre for Biological Sciences (TIFR), Bellary Road, Bangalore, Karnataka 560065, India

## Abstract

Sequence alignments are models that capture the structural, functional and evolutionary relationships between proteins. Structure-guided sequence alignments are helpful in the case of distantly related proteins with poor sequence identity, thus rendering routine sequence alignment methods ineffective. Protein Alignment organized as Structural Superfamilies or PASS2 database provides such sequence alignments of protein domains within a superfamily as per the Structural Classification of Proteins extended (SCOPe) database. The current update of PASS2 (i.e. PASS2.7) is following the latest release of SCOPe (2.07) and we provide data for 14 323 protein domains that are <40% identical and are organized into 2024 superfamilies. Several useful features derived from the alignments, such as conserved secondary structural motifs, HMMs and residues conserved across the superfamily, are also reported. Protein domains that are deviant from the rest of the members of a superfamily may compromise the quality of the alignment, and we found this to be the case in ∼7% of the total superfamilies we considered. To improve the alignment by objectively identifying such ‘outliers’, in this update, we have used a *k*-means-based unsupervised machine learning method for clustering superfamily members, where features provided were length of domains aligned, C^α^-RMSD derived from the rigid-body superposition of all members and gaps contributed to the alignment by each domain. In a few cases, we have split the superfamily as per the clusters predicted and provided complete data for each cluster. A new feature included in this update is absolutely conserved interactions (ACIs) between residue backbones and side chains, which are obtained by aligning protein structure networks using structure-guided sequence alignments of superfamilies. ACIs provide valuable information about functionally important residues and the structure–function relationships of proteins. The ACIs and the corresponding conserved networks for backbone and sidechain have been marked on the superimposed structure separately.

**Database URL:**

The updated version of the PASS2 database is available at http://caps.ncbs.res.in/pass2/.

## Introduction

Studying the protein sequence and structure space and understanding their link to protein function has been one of the key questions in the field of biology. In the case of proteins that resemble each other in terms of structure, but are sequentially distant, a comparison of the mere structures may not reveal much. Therefore, sequence alignments of such proteins serve as a useful evolutionary model. Unfortunately, the alignment of proteins with common evolutionary descent is not easy through routine multiple sequence alignment methods owing to poor sequence identity. For alignment of members of a superfamily—evolutionarily related, structurally similar yet sequentially divergent proteins—structure-based sequence alignment methods are more appropriate. These structure-guided sequence alignments may be used to perform rigid-body superposition of the proteins, which in turn may be used for structural identification of newly discovered proteins that are yet to find membership in a structural fold. The alignments themselves may be converted into hidden Markov models (HMMs) (1), which can be used for searches for remote homologues. Identification of residues that remain well conserved for all or most members provides cues about functionally important residues that may be used for identification of single nucleotide polymorphisms, mutational analyses and so on. Since proteins in a superfamily perform a similar function and are believed to descend from a common evolutionary ancestor, it will be useful to study the absolutely conserved residues (ACRs) and their network of interactions. Understanding this core architecture may assist in recognizing the function or assigning a superfamily for new members, and it also assists in designing proteins for a specific function.

Protein Alignment organized as Structural Superfamilies (PASS2) is a database that provides structure-based sequence alignments of protein domains within a SCOPe superfamily (2–7). With each update of SCOPe, PASS2 not only includes structure-based sequence alignments and associated features of newer domains and superfamilies but also introduces new features. The features in previous versions had included rigid-body superimposed structure of superfamily members, HMMs constructed based on structure-guided sequence alignments (1), list of residues conserved across all or most members, sequence motifs derived from the alignment, Gene Ontology (GO) terms for each protein domain and so on (8).

Within a superfamily, there may be members that have structural elements unique to themselves. This could be due to insertions within the protein domain resulting in a sequence longer than other members, and the additional residues may constitute secondary structural elements, which make the domain deviant from other members in the superfamily. In our last update, we had treated such structurally highly deviant members as outliers and not included these in the alignment (6). In this update, we have identified members that may be giants or dwarves (resulting in overhangs in the alignment or a large number of gaps) or structurally deviant from the rest of the members [based on C^α^-root-mean-square deviation (RMSD)] and using an unsupervised machine learning method, handled such domains on a case-by-case basis, to improve the quality of the alignment. An improved alignment results in higher confidence in the HMMs and other features derived out of it.

In addition to the existing features, in this update, we have included absolutely conserved interactions (ACIs) for each superfamily. Protein structure networks (PSNs) constructed for each member of the superfamily considering backbone and side chain atoms are aligned using structure-guided sequence alignment of the superfamily members, and common edges are identified as ACIs for that particular superfamily. These ACIs are plotted on superimposed structures of all members of the superfamily and interactive 3D visualization is available for each type of interaction (backbone, side chain and the ones common to both); the PyMol session files are also available for download from the database.

## Methods

The ASTRAL compendium within the SCOPe database provides Protein Data Bank (PDB) files of protein domains within a superfamily that share no >40% sequence identity with each other (7). This data set was obtained from SCOPe 2.07 and the data organized as superfamilies, according to SCOPe records. The superfamilies were distributed into two classes: multi-member superfamilies (MMSs) and single-member superfamilies (SMSs; superfamilies in which members are highly identical in sequence), and the PDB files were first processed to remove heteroatoms, incomplete residues and ‘GLX’ (GLU/GLN ambiguous) and ‘UNK’ (Unknown) characters. An initial alignment was performed for these PDB files using the program Matt (Multiple Alignment with Translations and Twists) (9), and this alignment was used to derive equivalent regions or non-gapped sequence blocks aligned for all the domain structures, using the program JOY (10). A different version of JOY was also used to annotate the Matt alignment with structural features as well as identify solvent accessibility, hydrogen binding and other structural features for each of the protein domains. Using the equivalences identified from the initial alignment and a tree constructed based on structural dissimilarity, COMPARER was run for obtaining a structure-based sequence alignment (11). Equivalences identified from the COMPARER alignment were used for rigid-body superposition of C^α^ backbones of the protein domain structures using the MNYFIT program, within JOY (10, 12, 13).

In addition to extreme structural outliers encountered while aligning MMS members using COMPARER (6, 11), in this update, we encountered members that would deteriorate the quality of the alignment by their unique structural elements, either rendering them structurally deviant (structural outliers) or possessing large insertions (giant members). There were also cases of dwarves or domains which were much smaller than other members of the superfamily, and introduce large numbers of gaps in the alignment. In the current update, we have addressed this problem by the employment of unsupervised machine learning (*k*-means) for clustering with the elbow method to estimate the optimal number of clusters in a superfamily. The features given for clustering include the percentage of gaps contributed by each domain to the alignment, the C^α^ RMSD calculated by JOY and the length of the domain. Scikit-learn (a Python machine learning library) was used to implement the machine learning methods for each alignment, and the optimal numbers of clusters predicted and features used were plotted for each superfamily. The outliers could be recognized automatically using this approach, and hence, alignments were thus improved either by trimming the giant member or removing a dwarf domain or a structural outlier domain or by splitting the superfamily into two or more split superfamilies, as per the clusters realized. The quality of the alignment was assessed in two ways: identification of secondary structural content using an in-house program, ASSALIMAV (4), and the percentage of gaps contributed by each domain to the alignment.

The features associated with the alignment are as follows: HMM created for each superfamily using the hmmbuild module within the HMMER package (1, 14), secondary structural motifs identified using Smotif (15), ACR and highly conserved residue (HCR) (100 and 80–99% conserved across all superfamily members, respectively) identified from each superfamily alignment, statistics of the alignment (ALISTAT) and indel-related information (CUSP) (16). JOY program was used to annotate the alignment as well as each superfamily member’s PDB file with secondary structural information such as hydrogen bonding, solvent accessibility and so on. To describe how similar/dissimilar the superfamily members are, the average C^α^-RMSD-derived distance matrix, the sequence identity matrix, the average C^α^-RMSD value in Å and a structural dissimilarity tree are provided for each superfamily. The alignment-derived HMMs are also used to search for newly deposited remote homologues of SCOPe superfamily members in PDB. GO terms are listed for each member within a superfamily as well (8).

PSNs were created for each superimposed structure of superfamily members, at two levels, by considering atoms in the (a) backbone and (b) side chain. For the backbone networks, two residues were marked as interacting, when their C^α^ atoms were found within a distance of 7.5 Å. The backbone network was constructed with residues as nodes and interactions depicted as edges among these nodes. Edges in sidechain networks were constructed for residues having contact between any of their side-chain atoms (all atoms except N, C^α^, C and O) at a distance cut-off of 4.5 Å. Similar to backbone networks, residues were considered as nodes and interactions represented by edges. Networks thus constructed were aligned as per the COMPARER alignment, by inserting dummy nodes at the positions of gaps (17). An edge or an interaction was said to be conserved when it was found aligned between the nodes in the same position. ACIs were identified for each superfamily, for both backbone and side chain networks. Interactions, visualized on the superimposed structure of superfamily members as PyMol session files, are available for download and these are also available for interactive viewing through the JSmol plug-in. Octave was used for implementing alignment of PSNs and identification of ACIs and PyMol for creating ACI figures.

The data were organized as ASCII files, images and database tables. Python scripts were used to prepare the organized collection of the files. The database tables are implemented in the MySQL5.2 database engine. Python, BioPython and Flask micro frameworks have been used as the backend engine interacting with the web interface, database and data file collection. The web interface was implemented with the support of CSS, JavaScript, Ajax and JQuery along with HTML and Bootstrap framework. Alignment visualization panels were implemented using the in-house custom-made plug-in. JSmol, Raphael and jsPhyloSVG were used for implementing the visualization panels for displaying the molecular structure (including conserved interactions), phylogenetic tree. Various Python scripts, working at the backend, use Restful API calls to fetch details such as the GO annotations from external sources dynamically.

## Results

### PASS2.7 database and workflow

**Figure 1.**
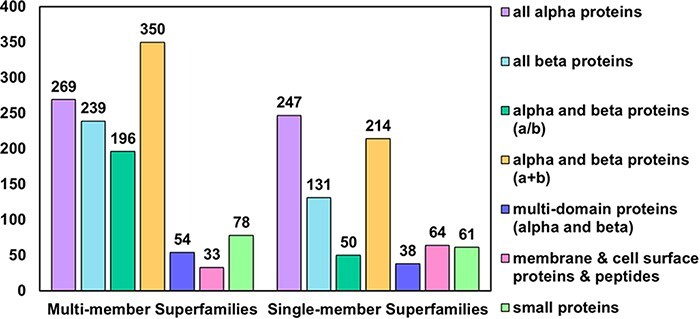
Distribution of MMS and SMS considered in PASS2.7 across the seven classes of SCOPe. The most represented class as in the case of MMS is that of alpha and beta proteins (a+b) followed by all-alpha proteins, while in the case of SMS, the reverse holds.

In the latest version of the PASS2 database, PASS2.7, a total of 2024 superfamilies have been considered, of which 1219 are MMSs and 805 are SMSs. These superfamilies contained a total of 14 323 SCOPe domains. SCOPe groups superfamilies into 12 classes, of which 7 are available for download from the ASTRAL compendium. The numbers of superfamilies (both SMS and MMS) from each of these seven classes were counted and have been represented in Figure 1. As observed in the previous versions of PASS2, the number of MMSs from the class alpha and beta proteins (a+b) are the highest in number compared to other classes, and the highest number of SMSs is from the all-alpha proteins class (Figure 1). The increments in the number of superfamilies and the number of SCOPe domains over the past few updates of the PASS2 database have also been depicted in Supplementary Figure 1.

### Improvement of alignment and automation of alignment protocol

**Figure 2.**
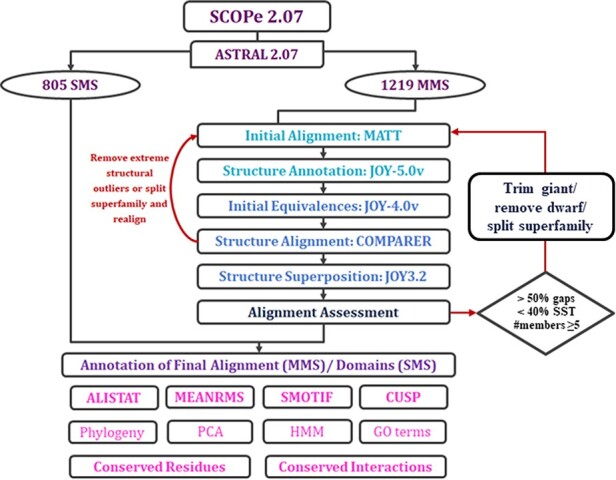
Workflow of PASS2.7 alignment and/or annotation of superfamily member(s). The newly added alignment improvement step involves the identification of MMS with at least five members whose alignments possess >50% gap positions and <40% conservation of secondary structural elements. Clusters are identified for such cases based on a machine learning algorithm, on basis of which the alignment is improved. The additional feature available for MMS in this update is the conserved interactions.

The alignment of protein domains within MMS in PASS2 consists of several steps that can be grouped into four main phases: organization of data, initial alignment, final alignment and annotation of the final alignment with several features (Figure 2). In this update, we have tried to automate the final alignment phase through the identification of errors imposed by different programs used, outliers and the points of possible manual intervention.

The final alignment produced by COMPARER may retain a large number of gaps that may have been incorporated while trying to align one or more member(s) that are different from the remaining members. Such difference may be brought about by structural dissimilarity (structural outlier) or length of the domain (dwarf or giant domain). We identified 87 MMSs that had >50% gaps contributed by at least one member in the alignment and conserved secondary structural content of <30%. For these MMSs, an unsupervised machine learning-based clustering method was used to identify the best possible way to improve the alignment.

**Figure 3.**
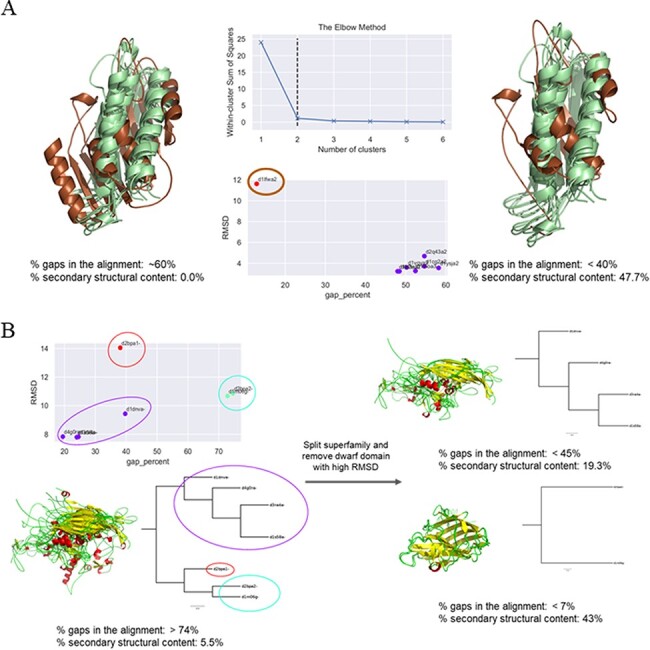
Examples of clustering method-based improvement of alignment. (A) For the superfamily bacterial exopeptidase dimerization domain (SCOPe ID 55031), the domain d1lfwa2 (brown) was found to be a giant member, which had RMSD >10 Å to other members and this led to a large number of gaps in the alignment. The overhang region of this domain was trimmed and the members realigned. This significantly improved the secondary structural conservation of the alignment and reduced the number of gaps. (B) Three clusters were predicted for the MMS ssDNA viruses (88645), and the alignment was found to be highly ‘gappy’ (>70% gaps). The MMS was split into two, and a dwarf domain that was the lone member of the third cluster was removed to obtain an improved alignment with a significantly lesser number of gaps in alignment of each split MMS and higher secondary structural conservation.

In cases where a clear outlier—in terms of either percentage of gaps or C^α^-RMSD or both—could be identified, this member was inspected for its domain length. If it was found to be a dwarf domain, the member was removed and the remaining members re-aligned to obtain a lower number of gaps and a higher number of conserved secondary structural elements. If the domain was found to be a giant member, the domain was ‘trimmed’ guided by the COMPARER alignment, *albeit* a ‘gappy’ (containing a large number of gaps) one, and re-alignment was performed for the MMS (example illustrated in Figure 3A). The splitting of superfamilies was also guided by the clusters predicted for the improvement of alignment. There were a few cases where the number of clusters (of domains within the MMS) predicted was three, but close inspection revealed the presence of a single structural outlier as a third cluster, which was then removed to obtain two split MMSs and improved alignment statistics (exemplified in Figure 3B).

**Figure 4.**
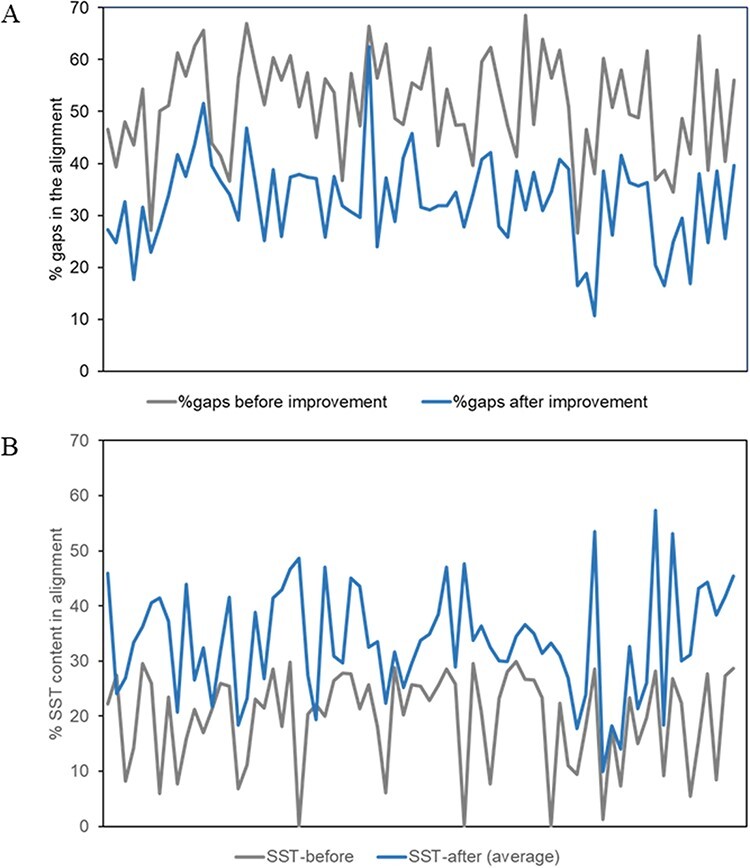
Plots of alignment statistics showing a decrease in the number of gaps in the alignment and an increase in conserved secondary structural positions. (A) Percentage of gaps, averaged over members of a superfamily, in the alignment before and after the improvement of alignment. (B) Equivalent secondary structural positions (SST) in the alignment of about 80 superfamilies, before and after the improvement of alignment.

The percentage of gaps (averaged over all members of the MMS) in the alignment and the conserved secondary structural content before and after the improvement of alignment for about 80 superfamilies, as described above, have been plotted in Figure 4.

**Figure 5.**
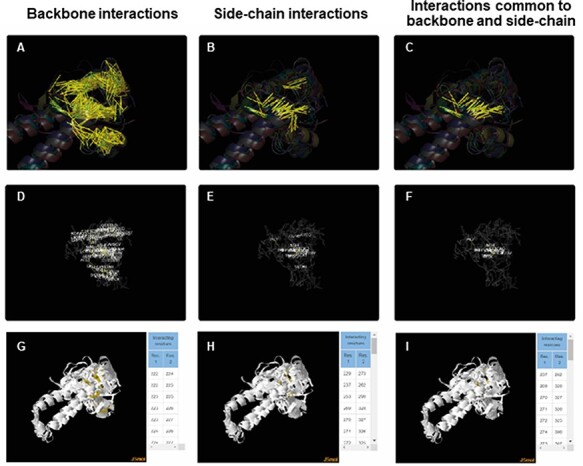
ACIs plotted on superposed structures for the Tubulin C-terminal domain-like superfamily (SCOPe ID 55307). The top panel (A–C) consists of figures showing ACIs on superposed structures for backbone, side chain and those common to both of them. The middle panel (D–F) consists of figures showing ACI networks with possible residues at respective positions on the representative structure of the superfamily. The bottom panel (G–I) shows the interactive views available for the three types of ACIs.

For each of the superfamily, ACIs were identified for backbone and side chain separately using PSNs as explained in the ‘Methods’ section. More details about the method of obtaining ACIs are available in the Help tab of the database. ACIs, common to both backbone and side chains, have also been computed and presented in the database. Figure 5 is a representation of the conserved interactions within the Tubulin C-terminal domain-like superfamily (SCOP ID 55307), selected as an illustration. ACRs and their conserved interactions can be appreciated in both the static (Figure 5A**–**F) and interactive images (Figure 5G**–**I). The PyMol session files used to generate these figures are downloadable individually or as a single zip file. The zip file also consists of the lists of ACIs and figures as .png files. Details about each file in the zip folder are enlisted in the Help tab.

### Other associated features of the alignment

Apart from the conserved interactions between backbone and side chains of residues of MMS members, several other useful features are provided within PASS2.7 for download. The statistics of the alignment (ALISTAT), information regarding length variation among MMS members due to insertions or deletions (CUSP) and conservation of secondary structural elements (ASSALIMAV) were provided alongside structural motifs (SMotif), conserved residues (ACR and HCR) for each alignment. The structure-based sequence alignments can be used to construct PSSMs, which can further be used for sensitive sequence searches, and the HMMs created out of these alignments provided in the database also serve this purpose. GO terms, PCA based on structural dissimilarity, as well as dissimilarity-based tree, provide information regarding the individual members of the MMS. For both SMS and MMS, structure and sequence information are amalgamated using JOY, which provides hydrogen bonding, solvent accessibility and secondary structural information for both the alignment (in case of MMS) and the domains (in both SMS and MMS).

## Conclusion

The availability of structure-based sequence alignments of 2024 protein domain superfamilies permits bioinformatic analyses, such as the construction of mathematical models for sensitive detection of additional members in the vast and ever-increasing space of protein sequences. They also serve as reliable evolutionary models for the comparison of incoming members, to design biochemical experiments and to appreciate structurally conserved/weak regions, insertions and deletions. The provision of accessory structural data, such as conserved structural blocks, structure-annotated derived files and the brand new feature of networks of conserved residues for the superfamilies, organized within this PASS2 database, should be useful for further structural and biochemical analyses. The identification of structural outliers using machine learning techniques, as introduced in this update, should enable rapid updates in future.

## Supplementary Material

baac025_Supp
