# Supplementary figures and images for: PASS2.7: a database containing structure-based sequence alignments and associated features of protein domain superfamilies from SCOPe
Source: Database (Oxford). 2022 Apr 12;2022:baac025. doi: 10.1093/database/baac025 (PMC9216583; doi:10.1093/database/baac025)

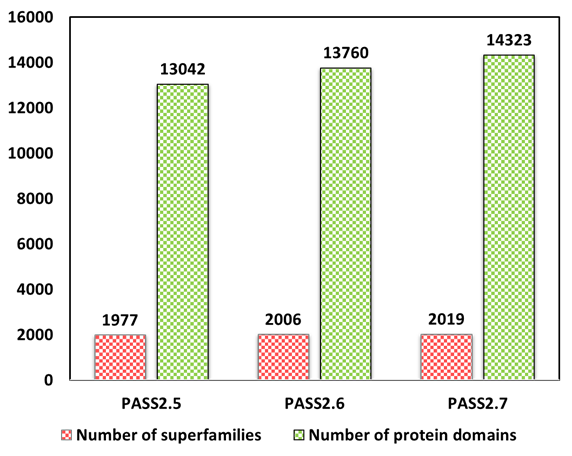

Supplement: baac025_Supp [file baac025_supp.zip › supplfig1_72dpi.tif]
